# Comprehensive analysis of lncRNAs and mRNAs revealed potential participants in the process of avian reovirus infection

**DOI:** 10.3389/fmicb.2025.1539903

**Published:** 2025-02-05

**Authors:** Shaqiu Zhang, Jinkang Li, Mingshu Wang, Renyong Jia, Shun Chen, Mafeng Liu, Dekang Zhu, Xinxin Zhao, Ying Wu, Qiao Yang, Juan Huang, Xumin Ou, Di Sun, Bin Tian, Yu He, Zhen Wu, Anchun Cheng

**Affiliations:** ^1^Avian Disease Research Center, College of Veterinary Medicine, Sichuan Agricultural University, Chengdu, China; ^2^Institute of Veterinary Medicine and Immunology, Sichuan Agricultural University, Chengdu, China; ^3^Key Laboratory of Animal Disease and Human Health of Sichuan Province, Sichuan Agricultural University, Chengdu, China; ^4^Engineering Research Center of Southwest Animal Disease Prevention and Control Technology, Ministry of Education of the P.R. China, Chengdu, China

**Keywords:** avian reovirus, transcriptome sequencing, long noncoding RNA, *linc000889*, avian reovirus replication

## Abstract

Avian reovirus (ARV), a double-stranded RNA virus, frequently induces immunosuppression in poultry, leading to symptoms such as irregular bleeding and spleen necrosis in infected ducks. Since 2017, the morbidity and mortality rates associated with ARV infection in poultry have been on the rise, progressively emerging as a significant viral disease impacting the duck farming industry in China. In our study, we collected duck embryo fibroblasts 18 h post-infection with ARV and conducted transcriptome sequencing analysis. The analysis revealed that 3,818 mRNA expressions were up-regulated, 4,573 mRNA expressions were down-regulated, 472 long noncoding RNAs (LncRNAs) were up-regulated, and 345 lncRNAs were down-regulated. We employed qRT-PCR to validate the sequencing results, confirming their accuracy. The transcriptome data indicated significant upregulation of the *PARP9*, *TLR7*, *TRIM33*, and *ATG5* genes, suggesting their potential involvement in ARV infection. Notably, our study identified a novel functional lncRNA, *MSTRG.9284.1* (It was named *linc000889* in the present study), which inhibits the replication of ARV at the transcriptional, translational levels and viral titer. Overall, this study has identified numerous ARV-induced differentially expressed mRNAs and lncRNAs, including the functional lncRNA *linc000889* that inhibits ARV replication. This discovery provides new insights into the mechanisms of ARV infection and may contribute to the development of new prevention and treatment strategies.

## Introduction

Avian Reovirus (ARV) is a significant viral pathogen that is extensively prevalent in poultry, leading to respiratory, gastrointestinal, and joint diseases (Ayalew et al., 2017). For instance, several duck species exhibit symptoms such as a loss of appetite, gait instability, diarrhea, and death (Mase et al., 2021). ARV is classified within the *genus Orthoreovirus* of the family *Reoviridae* and is as prevalent as other traditional epidemic diseases, including avian influenza, duck plague, and duck Tembusu virus (Fang et al., 2024). During the peak of disease outbreaks, ARV can induce secondary infections due to immunosuppression, thereby posing a serious threat to the duck industry in China. The ARV genome is divided into three groups, comprising 10 segments of double-stranded RNA. The S group segments (*S1*, *S2*, *S3*, *S4*) encode six proteins: *S1* gene encodes for p10, p17, and σC, while *S2*, *S3*, and *S4* genes encode for σA, σB, and σNS proteins, respectively. The L group segments (*L1*, *L2*, and *L3*) encode λA, λB, and λC proteins, respectively. Additionally, the M group segments (*M1*, *M2*, and *M3*) encode μA, μB, and μNS proteins, respectively (Egana-Labrin and Broadbent, 2023). Among them, the σC protein significantly influences virus adsorption and proliferation. Specifically, it recognizes and binds to receptors located on target cells, thereby initiating the process of viral invasion (Lostalé-Seijo et al., 2016; Huang et al., 2022). Therefore, we selected the level of σC protein expression to evaluate ARV replication.

Long non-coding RNA (LncRNA) is a class of regulatory non-coding ribonucleic acids with fragment sizes ranging from 200 bp to 100 kb that do not encode proteins (Ferrè et al., 2016). A mere 2% of genes are translated into messenger RNA. A significant number of non-coding RNAs (nc RNAs) generated during transcription have historically been regarded as “transcriptional noise,” lacking clear biological functions (Qian et al., 2019). However, with the continual advancement of high-throughput sequencing technology, an increasing number of lncRNAs have been identified, thereby laying the foundation for functional studies of lncRNAs. Moreover, previous studies on the involvement of nc RNAs in virus-host interactions have mainly focused on small nc RNAs (e.g., microRNAs), but the biological functions played by lncRNA in virus-host interactions have gradually aroused widespread attention (Cui et al., 2020; Mattick et al., 2023). LncRNAs influence viral replication by modulating the host immune response, impacting epigenetic modifications, interacting with viral genes, recruiting host factors, and stabilizing viral RNA (Atianand et al., 2016; Fernandes et al., 2019; Wang et al., 2021). In hepatitis B virus (HBV) infection, the host lncRNA *HULC* can functions as a sponge for *microRNA-372*, thereby preventing *microRNA-372* from inhibiting Nuclear Factor-κB (NF-κB). This mechanism promotes HBV replication and facilitates immune evasion (Yu et al., 2017). The lncRNA *LAT* induced by Herpes simplex virus type 1 (HSV-1) can bind to the HSV-1 genome within human neurons. This binding facilitates the relocation of the HSV-1 genome to the perinuclear space, fostering the establishment of viral latency, and ultimately modulates viral replication (Grams Tristan et al., 2023). Understanding these mechanisms can provide references and ideas for us to study the effects of host lncRNA on ARV replication.

Currently, lncRNAs have emerged as a focal area in scientific research, and the threat posed by ARV to the duck farming industry in China cannot be overlooked. However, research on duck lncRNAs remains relatively limited. To establish a scientific foundation for ARV control strategies from the perspective of lncRNAs, we employed transcriptome sequencing technology to identify lncRNAs that may impact ARV replication. The sequencing results successfully identified a multitude of differentially expressed (DE) mRNAs and lncRNAs, indicating substantial research potential. Notably, we screened a novel duck lncRNA, *MSTRG.9284.1* (*linc000889*), which exhibited a significant inhibitory effect on ARV replication. In the future, it is expected to provide more basis for understanding or preventing ARV infection at the molecular biological level.

## Materials and methods

### Cells and virus

In this study, all experiments were conducted using duck embryo fibroblasts (DEF), which were prepared from 10-day-old duck embryos purchased from a duck farm in Ya’an, Sichuan province. Their tissues undergo trypsin digestion, and the resultant cells are subsequently inoculated into a high-glucose medium enriched with 10% fetal bovine serum (FBS, Gibco, United States) and supplemented with 2% penicillin–streptomycin solution (Beyotime, China). Cultivation proceeds under optimal conditions of 37°C and 5% CO_2_. The viral titer of the ARV strain (BioSample: SAMN00173207) was 10^–5.5^ 50% tissue culture infective does (TCID_50_)/100 μL. After a 1-h adsorption period, wash the cells three times with PBS, then add DMEM medium containing 2% FBS to continue culturing the cells. The experiments were repeated three times for each group. Uninfected cells were used as a control group. Cell samples were collected 18 h post-infection, following treatment with RNAiso Plus (Takara, Japan), and were then sent to Shanghai Personal Biotechnology Co., Ltd. for transcriptome sequencing. The experimental groups were designated as DEF-V1, DEF-V2, and DEF-V3, whereas the control groups were named DEF-C1, DEF-C2, and DEF-C3.

### Identify differentially expressed genes

The quality assessment revealed that all sample RIN values were perfect at 10, satisfying the criteria for library preparation and sequencing. We use Cutadapt to process raw data to eliminate low-quality reads and Trimmomatic (v.0.38) to further refine the dataset and retain only high-quality sequences. These high-quality reads were then aligned with the reference duck genome (Anas_platyrhynchos.ASM874695v1.dna.toplevel.fa) using the HISAT 2 tool,^1^ and tools like Protein coding potential of long non-coding RNAs by Kmer feature (PLEK), Coding-Non-Coding Index (CNCI), and Pfam protein family database scan (PfamScan) were employed for further analysis. The filtered reads were mapped onto the duck genome, and PLEK was utilized to perform a principal component analysis (PCA) of the expression levels across samples with the DESeq software package.

To standardize the comparison of gene expression, we adopted the Fragments Per Kilobase of exon model per Million mapped fragments (FPKM) method within the Cufflinks program as our metric. Additionally, Stringtie was employed to ascertain lncRNA reads, which were then analyzed for expression level homology using FPKM. Differentially expressed genes (DEGs) were identified based on the criteria of |log_2_ fold change (DEF-C/DEF-V) | > 1 and *p* < 0.05.

### Target genes prediction of lncRNAs and competitive endogenous RNAs regulatory network construction

To elucidate the diverse functions of lncRNAs, we distinguished between cis- and trans-acting mechanisms. Cis-target genes, potentially regulated by lncRNAs, were identified within a 100 kb window around the lncRNA genes (Dhaka et al., 2024). These genes may interact with nearby cis-acting elements or co-expressed genes to modulate transcriptional or post-transcriptional gene expression. In contrast, trans-acting lncRNAs are associated with co-expressed protein-coding genes, regardless of their physical location. We identified trans-regulatory links between lncRNAs and mRNAs using a stringent correlation threshold of |correlation| > 0.95 and a *p*-value of <0.05.

For the construction of a ceRNA regulatory network, we utilized miRanda software to predict target miRNAs for our lncRNAs, based on duck-encoded miRNA sequences. This process yielded miRNA-lncRNA interaction pairs.

### *In-silico* gene functional prediction analysis

We performed Gene Ontology (GO) enrichment analysis on DEGs and their targets using TopGO, leveraging the GO database^2^ for annotations. The significance of GO terms enrichment was determined by hypergeometric distribution with a *p*-value cutoff of 0.05. Additionally, we utilized the Kyoto Encyclopedia of Genes and Genomes (KEGG^3^) to predict pathways associated with DEGs and targets, applying the same significance threshold. KAAS and eggnog-mapper were then used to annotate the identified GO and KEGG pathways, respectively.

### qRT-PCR analysis

We utilized the RNAiso Plus reagent (TaKaRa, Japan) to efficiently extract total RNA from cells. Subsequently, we determined the concentration and purity of the extracted RNA using a NanoDrop 2000 spectrophotometer (Thermo Fisher Scientific, United States). The RNA was then reverse-transcribed into cDNA using the Hifair III 1st Strand cDNA Synthesis SuperMix for qPCR kit (Yeason, China). Following the protocol of the ChamQ SYBR mix kit (Yeason, China), we performed quantitative analysis of target gene RNA expression using Bio-Rad CFX96 Real Time Detection System (Bio-Rad, United States). The sequences of the gene-specific primers used in the experiment are detailed in Table 1. For this study, we selected *GAPDH* as the internal reference gene to ensure the accuracy and reliability of our experimental results. Employing the 2^−ΔΔCt^ method, we calculated the relative expression levels of each target RNA. Each experiment conducted in triplicate.

**Table 1:** The primers used in the present study.

Gene names	Forward (F) or Reverse (R)	Sequences (5′-3′)
ENSAPLG00020006869 (RAG1)	F	GGTCTTCCACTCCATCACCA
	R	AACTCCGTTGCATTGCCAAT
ENSAPLG00020005552 (THEMIS)	F	CAAGAGTCCTAGCCTCCGAG
	R	ACGACATGAAGCTGTTCCCT
ENSAPLG00020001201 (CCDC3)	F	GATGGGTGTGAAGGAAGTGC
	R	AAGCTTGGGGTCATGATGGA
ENSAPLG00020003815 (USF1)	F	GCGTGGAGATCGTCATCAAG
	R	GGAGGAGGCACAAACCAAAC
MSTRG.10330.1	F	TTTCCCTTCCTCACCTCGTC
	R	GAGGTGTTTGTCCCAAACCC
MSTRG.15824.2	F	TCCAAGGACTCACAGCTTCC
	R	GTCTGTGGCTACTGTCCTCA
MSTRG.3297.7	F	AAAAGCCACGCCAAAGACAT
	R	TCCATCAGCCAAGGGAATGT
MSTRG.7914.2	F	CGTTCTGGCCCATTCACAAA
	R	AAGGGAACAATTGGCTGCAG
ARV-S1	F	CCTTGTCTCCGATCCTCTCC
	R	CAATGGCAGGGGTCGTTATG
linc000889 (qPCR)	F	ATCCCACAAGCTCCCAAAGA
	R	GGCTTTGCTCAGTTCTGCTT
PCA-linc000889	F	CATCATTTTGGCAAAGAATTCAATTCTGGAATTTCCACTTGGGCT
	R	TTGGCAGAGGGAAAAAGATCTTGTTTTATCCAAATTCTTTATTCTCCAGAATCATAATT
PEGFP-linc000889	F	GTCCGGCCGGACTCGCAGATCTAATTCTGGAATTTCCACTTG
	R	AACCTAGTATAGGGGAGAATTCTGTTTTATCCAAATTCTTTATTCTCC
U6	F	CTCGCTTCGGCAGCACA
	R	GCGTGTCATCCTTGCGC
GAPDH	F	GCAGATGCTGGTGCTGAATA
	R	TCATGGTTCACACCCATCAC
ShRNA-159		TTCTCGCTCTTCTCCTCATTG
ShRNA-665		ATGGGACTGCGCCTCTATTGT
ShRNA-802		GGCTCAGAGTCTCCGAACAGGA

### Plasmid construction and transfection

We amplified the full-length *linc000889* fragment based on raw data obtained from transcriptomics. The successfully amplified full-length fragment of *linc000889* was then inserted into the PCAGGS vector (Addgene, United States) using homologous recombination (Vazyme, China). Thus, we successfully obtained the experimental plasmid PCA-linc000889 and the control plasmid PCAGGS. Similarly, we obtained the plasmid PEGFP (Addgene, United States) and PEGFP-linc000889. We also commissioned GenePharma (China) to synthesize shRNA targeting *linc000889*. From this, we obtained three shRNAs targeting *linc000889* expression: PGPU6-shRNA-159, PGPU6-shRNA-665, and PGPU6-shRNA-802, as well as the control shRNA PGPU6. DEF were transfected using Lipofectamine 2000 (Yeason, China) after being plated in a 12-well plate for 12–24 h, at which point the cell density reached approximately 80%.

### Virus titer determination

Samples were collected at various time points, and the cells underwent two freeze–thaw cycles. The virus sample was then centrifuged and stored at −80°C. Subsequently, virus suspension samples were diluted in a 10-fold serial dilution ranging from 10^−1^ to 10^−8^. Each dilution was dispensed into eight replicate wells of a 96-well cell culture plate, with 100 μL of diluted viral suspension added per well. Following this, 100 μL of a passaged cell suspension was added to each well, and the plates were incubated at 37°C with 5% CO₂ for 5–7 days. Cytopathic effects (CPE) were observed and documented during this period. The viral titer, expressed as TCID_50_, was calculated using the Reed-Muench method. Each experiment conducted in triplicate.

### Western blot

We lysed cell cultures at 4°C using a RIPA lysis buffer (Beyotime, China), supplemented with 1 mM PMSF (Beyotime, China), to extract proteins. The denatured protein samples were resolved by SDS-PAGE and transferred onto 0.45 μm PVDF membranes (Millipore, United States). After blocking the membranes in 5% skim milk in Tris-buffered saline with tween-20 (TBST) for 2 h, we washed them with TBST containing 0.1% Tween 20. Following the manufacturer’s protocol, we incubated the membranes with primary antibodies diluted appropriately overnight at 4°C: anti-ARV-σc antibody (Prepared in this study, 1:1,000), anti-GAPDH antibody (Proteintech, China, 1:5,000), and anti-GFP antibody (Beyotime, China, 1:2,000). After incubation, the membranes were washed three times with TBST and then incubated with goat anti-mouse or anti-rabbit secondary antibodies (Abclonal, China, 1:10,000) at 37°C for 60 min. Subsequent washing was followed by treatment with ECL reagents (Thermo Fisher Scientific, United States). Proteins bound to the membrane were visualized and analyzed using the Touch Imager (e-BLOT, China). GAPDH served as the internal reference protein, with each experiment conducted in triplicate. Image J software was utilized for grayscale analysis to assess the differential expression of the proteins of interest.

### Separation of cellular components

After collecting DEF samples, we strictly followed the operational manual of the PARIS™ kit (Thermo Fisher Scientific, United States) to fractionate the cellular components, isolating pure cytosolic and nuclear fractions. We then extracted RNA from these distinct compartments and performed qRT-PCR for quantitative analysis. Each experiment conducted in triplicate. In this process, *U6* was used as the internal reference gene for the nuclear fraction, while *GAPDH* served as the internal reference gene for the cytosolic fraction.

### Cell viability assay

We employed the Cell Counting Kit-8 (CCK-8) (Yeason, China) to ascertain the cell viability with precision. The experimental outcomes were expressed as follows: the viability rates of the various treatment groups were presented as relative percentages in comparison to the control cells, which were designated a 100% viability rate. Each experiment conducted in triplicate.

## Results

### Characterization of ARV infection in DEF

**Figure 1:**
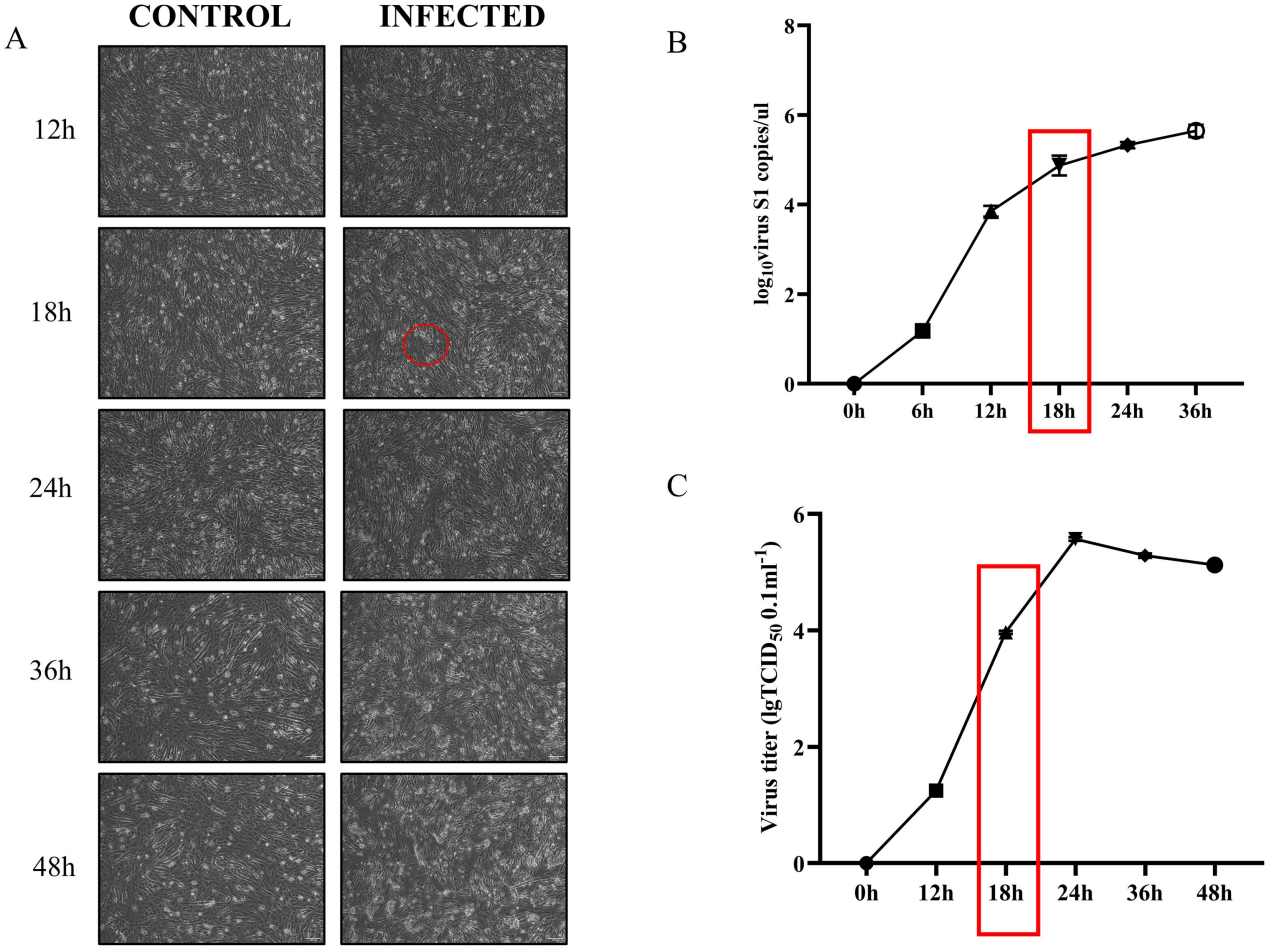
ARV infection of DEF at an MOI of 1 leads to progressive cytopathic effects and increased viral proliferation over time. **(A)** Cell lesions in DEF infected with ARV compared to uninfected controls at various time points. The red circle highlights the onset of cytopathic effects at 18 h, characterized by blurred cell boundaries and loss of fibrous morphology (Picture magnification is 100×). **(B)** Changes in ARV-S1 gene copy number in virus-infected and uninfected control groups over time. The red box indicates a continued increase in S1 gene copy number at 18 h. **(C)** Viral titer changes in virus-infected and uninfected control groups over time. The red box indicates an ongoing increase in viral titer at 18 h.

We established a model of ARV-infected DEF by infecting DEF with multiplicity of infection (MOI) of 1.0 ARV. In our study, DEF inoculated with ARV exhibited CPE at 18 h post-infection, as indicated by the red circle in Figure 1A, these represent cells whose structure and shape begin to become irregular, losing their original fibrous form. Over time, cell boundaries become increasingly indistinct, and cells begin to shed (Figure 1A). The viral titers of ARV per 100 μL were determined using the TCID_50_ assay. To ascertain the copy number of the ARV genome, we employed qRT-PCR, using the specific primer sequences outlined in Table 1. Subsequently, the corresponding viral copy numbers were calculated using a standard curve (Supplementary Table S1) that we prepared in this study. The results demonstrated that at 18 h post-infection, both the viral copy number (Figure 1B) and the viral titer (Figure 1C) were on the rise, albeit they had not yet attained their peak levels. Therefore, we chose 18 h and a MOI of 1.0 to prepare cell samples for future high-throughput RNA sequencing analysis.

### Transcriptome sequencing data analysis and screening

After rigorous filtering, RNA sequencing (RNA-seq) yielded an average of 72,300,972 and 79,255,743 clean reads in the ARV-infected group and control group, respectively. The control group had reads that were mapped to the reference genome ranging from 66,311,178 to 78,077,252, while the ARV-infected group had unique mapped reads ranging from 43,507,884 to 52,519,464 (Supplementary Table S2). Specific raw data can be found in the NCBI database PRJNA1145885. According to the results, the sequencing data had good quality and satisfied the requirements of the follow-up test with Q20 > 97% and Q30 > 93%. According to mRNA expression, PCA analysis revealed that the six samples’ principal components were dispersed within a 99% confidence interval (Supplementary Figure S1A). Nonetheless, the total lncRNA of the six samples differed more than the mRNA did, and its PCA distribution fell within the 31% confidence interval (Supplementary Figure S1B).

### Coding potential analysis and differential expression analysis

**Figure 2:**
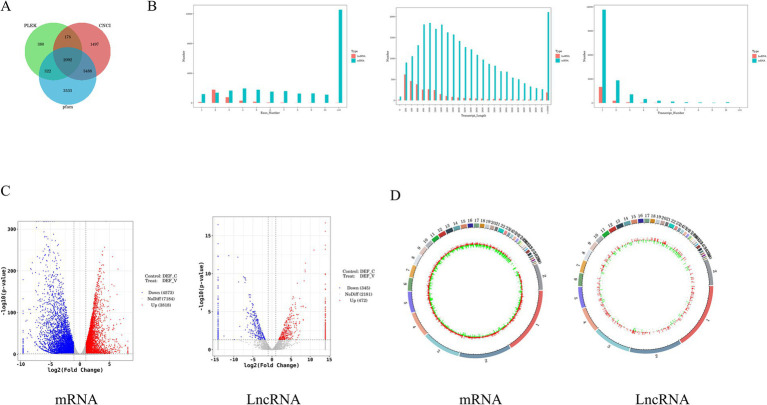
Differential analysis of lncRNA and mRNA expression in DEF infected with ARV. **(A)** Transcript Venn diagram without coding potential. **(B)** Comparison of the number, length, and number of transcripts between lncRNA and mRNA. **(C)** The volcano plot of the DE mRNAs and DE lncRNAs in DEF infected with ARV. **(D)** Differentially expressed mRNA and lncRNA circles of markers on the duck genome.

To examine the possibility of coding in candidate lncRNAs, we used three techniques: PLEK, CNCI, and PfamScan (Wang et al., 2010; Li et al., 2014; Love et al., 2014). A total of 2,092 high-confidence lncRNAs were found in the results and were then the topic of additional investigation (Figure 2A). We performed a structural comparison between known mRNAs and novel and known lncRNAs, concentrating on transcript count, length, and exon count. Comparing the number of transcripts in lncRNAs and mRNAs, we found that most lncRNAs have only 1–5 transcripts, while most mRNAs have 1–5 transcripts, with a smaller percentage having 6–10 transcripts. Length-wise, mRNAs have a more widely distributed length, with a notable proportion surpassing 5,000 nucleotides, whereas the majority of lncRNAs are between 200 and 1,200 nucleotides. Additionally, a comparison of exon counts showed that mRNAs frequently have more than 10 exons, which contributes to their higher capacity for protein coding, whereas most lncRNAs only have 1–5 exons (Figure 2B).

The selection of *p* < 0.5 and |log_2_FoldChange| > 1 as the screening criteria for differential genes between the experimental and control groups was chosen. We identified 8,391 DE mRNAs in DEF after ARV infection, of which 3,818 were up-regulated and 4,573 were down-regulated (Supplementary Table S3), and 817 DE lncRNAs, of which 472 were up-regulated and 345 were down-regulated (Supplementary Table S4). In addition, we used a volcano gram and heat map to display DE lncRNA and DE mRNA (Figure 2C). The genomic loop map revealed that LncRNA exhibited a greater degree of up-regulation across each chromosome, accompanied by a less uniform distribution of differentially expressed genes. In contrast, mRNA displayed a more even distribution of differentially expressed genes on each chromosome (Figure 2D). Among them, the mRNAs with significant differences in expression levels were *STING1*, *OASL*, *TRIM33*, *TLR7*, *PARP9*, *IFIH1*, *ATG5*, *CCL5*, *TBK1*, *TRIM29*, and *STAT1*. A number of *DUSP* genes, including *DUSP7*, *DUSP14*, *DUSP16*, *DUSP5*, *DUSP15*, *DUSP23*, *DUSP4*, and *DUSP26*, were noticeably down-regulated among them. And *DUSP19*, *DUSP28*, *DUSP12*, were noticeably up-regulated among them. In addition, the lncRNAs with significantly up-regulated expression levels were *MSTRG.7914.2*, *MSTRG.6760.1*, *MSTRG.2433.1*, *linc000889*, *MSTRG.12963.1*. Among them, the log_2_ fold values of *MSTRG.7914.2*, *MSTRG.6670.1*, and *linc000889* are −11.320, 6.116, and 10.524, respectively.

### Prediction of interacting genes and construction of ceRNA networks

**Figure 3:**
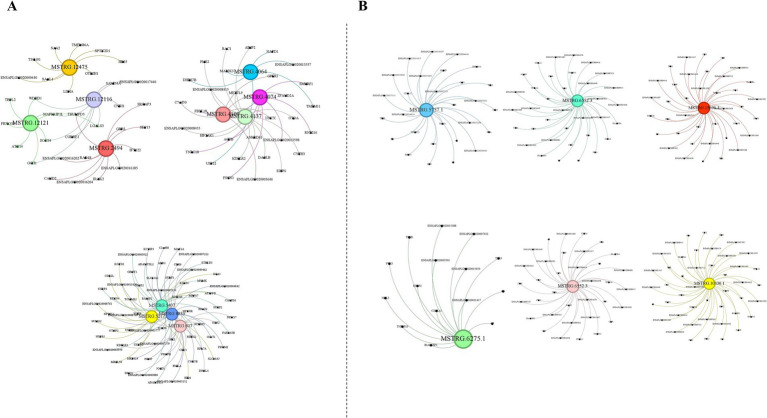
Prediction of potential cis-target and trans-target genes of lncRNAs. **(A)** Prediction of potential cis-target genes of lncRNAs. **(B)** Prediction of potential trans-target genes linked to immunity of lncRNAs. The images were created using the Gephi software.

In our study, we predicted the potential interactions of lncRNAs in both cis- and trans-regulatory modes. Our results indicated that the majority of lncRNAs harbored numerous cis-target genes, whereas each lncRNA typically interacted with thousands of trans-target genes. Subsequently, we randomly selected several lncRNAs and elucidated their cis-target genes (Figure 3A). Given the extensive number of lncRNA trans-target genes, we specifically focused on immune-related trans-target genes for display (Figure 3B).

Furthermore, we had projected the formation of competitive endogenous RNA (ceRNA) networks. The interplay between lncRNAs and miRNAs had been crucial for the regulation of gene expression. LncRNAs could serve as ceRNAs, inactivating miRNAs by binding to them and thus inducing a range of biological effects. Since lncRNAs were structurally analogous to mRNAs, miRNAs might have negatively regulated lncRNA expression through a mechanism similar to that affecting mRNAs. Additionally, miRNAs could enhance the expression of specific lncRNAs (Wang et al., 2022; An et al., 2023). In this study, we had obtained predictive outcomes for the interactions between differentially expressed lncRNAs and duck-encoded miRNAs. We had discovered that each miRNA was predicted to interact with a multitude of lncRNAs, with many lncRNAs being targeted by multiple miRNAs concurrently. For instance, in our study, we had predicted 8 miRNAs to target *MSTRG.5711.3*, while *apl-miR-11588-3p* was found to target 645 lncRNAs (Supplementary Table S5).

### Functional enrichment analysis of DE lncRNAs

GO and KEGG analyses were performed to predict the bioregulatory functions of host duck lncRNAs. DEGs were classified according to GO terms, including molecular functions, cellular components, and biological processes, to elucidate the potential roles of host factors involved in ARV infection.

**Figure 4:**
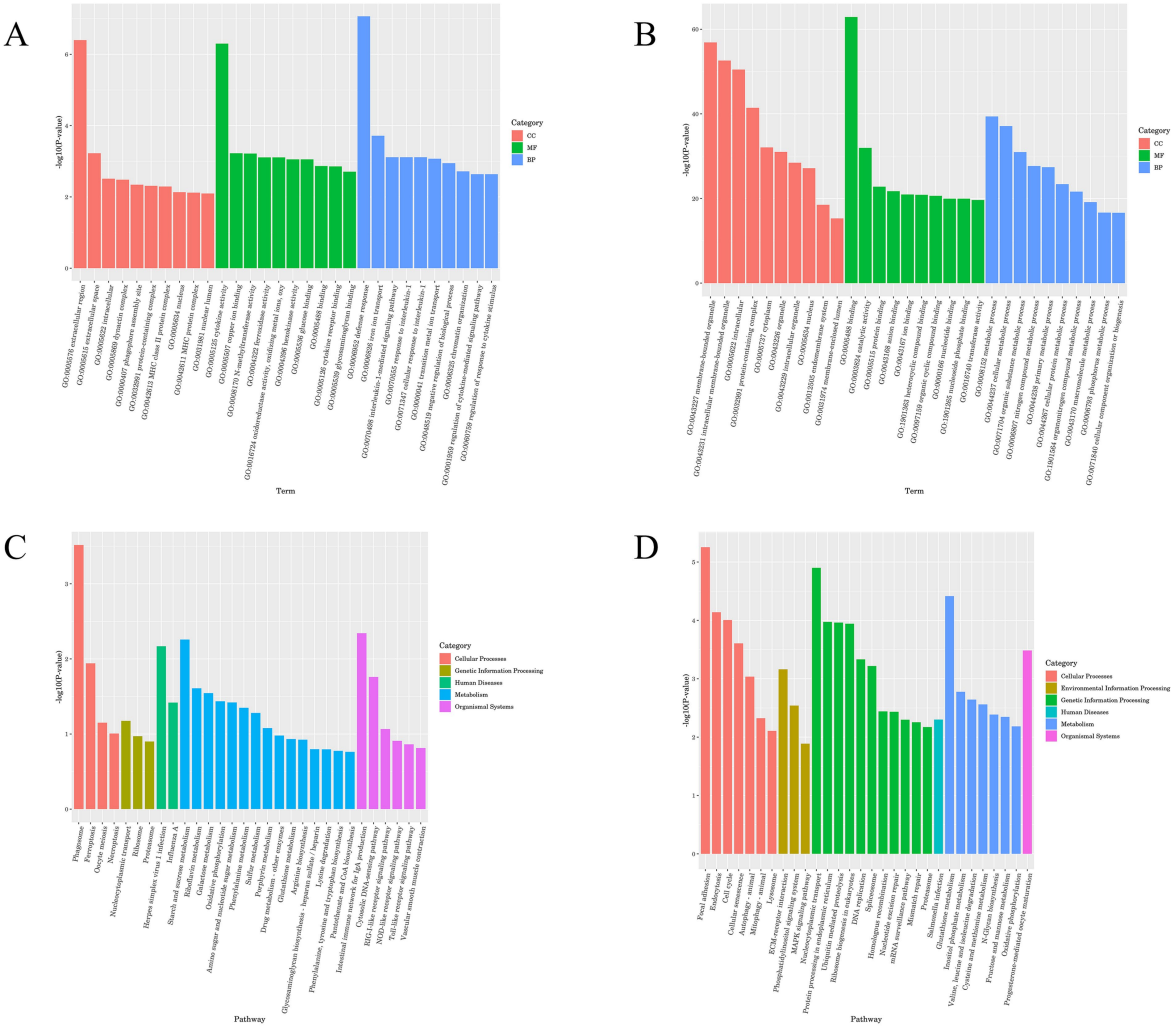
GO and KEGG enrichment map of lncRNA. **(A)** GO enrichment analysis histogram of lncRNA cis-target genes. **(B)** GO enrichment analysis histogram of lncRNA trans-target genes. **(C)** KEGG enrichment analysis histogram of lncRNA cis-target genes. **(D)** KEGG enrichment analysis histogram of lncRNA trans-target genes.

The most remarkable GO terms related to biological processes were the defense response (GO:0006952), iron ion transport (GO:0006826), metabolic process (GO:0008152), and cellular metabolic process (GO:0044237). Most of the DEGs in both the control and experimental groups were found to be related to cellular components, such as membrane−bounded organelle (GO:0043227), intracellular membrane−bounded organelle (GO:0043231), intracellular (GO:0005622), protein containing complex (GO:0032991), and extracellular region (GO:0005576). The majority of the DEGs were also involved in stimulus-related molecular functions, including binding (GO:0005488), catalytic activity (GO:0003824), cytokine activity (GO:0005125), and protein binding (GO:0005515) (Figures 4A,B).

Signaling pathway analysis contributes to a better understanding of the biological functions of genes, and KEGG pathway enrichment analysis of DEGs can be used to further facilitate our exploration of the mechanisms of host-virus interactions.

The DEGs were subjected to KEGG term categorization statistics involving Cellular Processes, Environmental Information Processing, Genetic Information Processing, Human Diseases, Metabolism, and Organismal Systems. The most remarkable KEGG related to Organismal Systems were the Intestinal immune network for IgA production, Toll−like receptor signaling pathway, Cytosolic DNA − sensing pathway, RIG−I − like receptor signaling pathway, and NOD−like receptor signaling pathway. The KEGG related to Cellular Processes were Phagosome, Cell cycle, Focal adhesion, Autophagy, and Mitophagy (Figures 4C,D).

### Functional enrichment results of DE mRNAs

**Figure 5:**
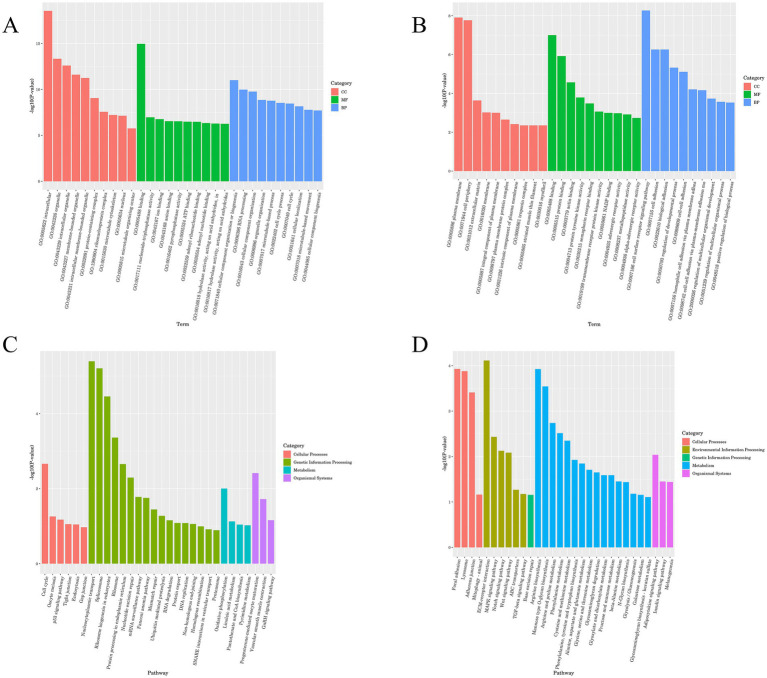
GO and KEGG enrichment map of mRNA. **(A)** GO enrichment analysis histogram of mRNA differentially up-regulated genes. **(B)** GO enrichment analysis histogram of mRNA differentially down-regulated genes. **(C)** KEGG enrichment analysis histogram of mRNA differentially up-regulated genes. **(D)** KEGG enrichment analysis histogram of mRNA differentially down-regulated genes.

In GO terms, the most remarkable GO terms related to biological processes were the organonitrogen compound metabolic process (GO:1901564), and cellular localization (GO:0051641), Most of the DEGs in the control group and the experimental group were related to cellular components, including protein−containing complex (GO:0032991). Most DEGs are assigned to stimulus-related molecular functions, including binding (GO:0005488), protein binding (GO:0005515), ion binding (GO:0043167), and anion binding (GO:0043168) (Figures 5A,B).

The most significant KEGG enrichment pathway was related to the ECM–receptor interaction, Focal adhesion, Mitophagy animal, Cysteine and methionine metabolism, Adipocytokine signaling pathway, and Endocytosis (Figures 5C,D).

### Validation of DE lncRNAs and mRNAs by qRT-PCR

**Figure 6:**
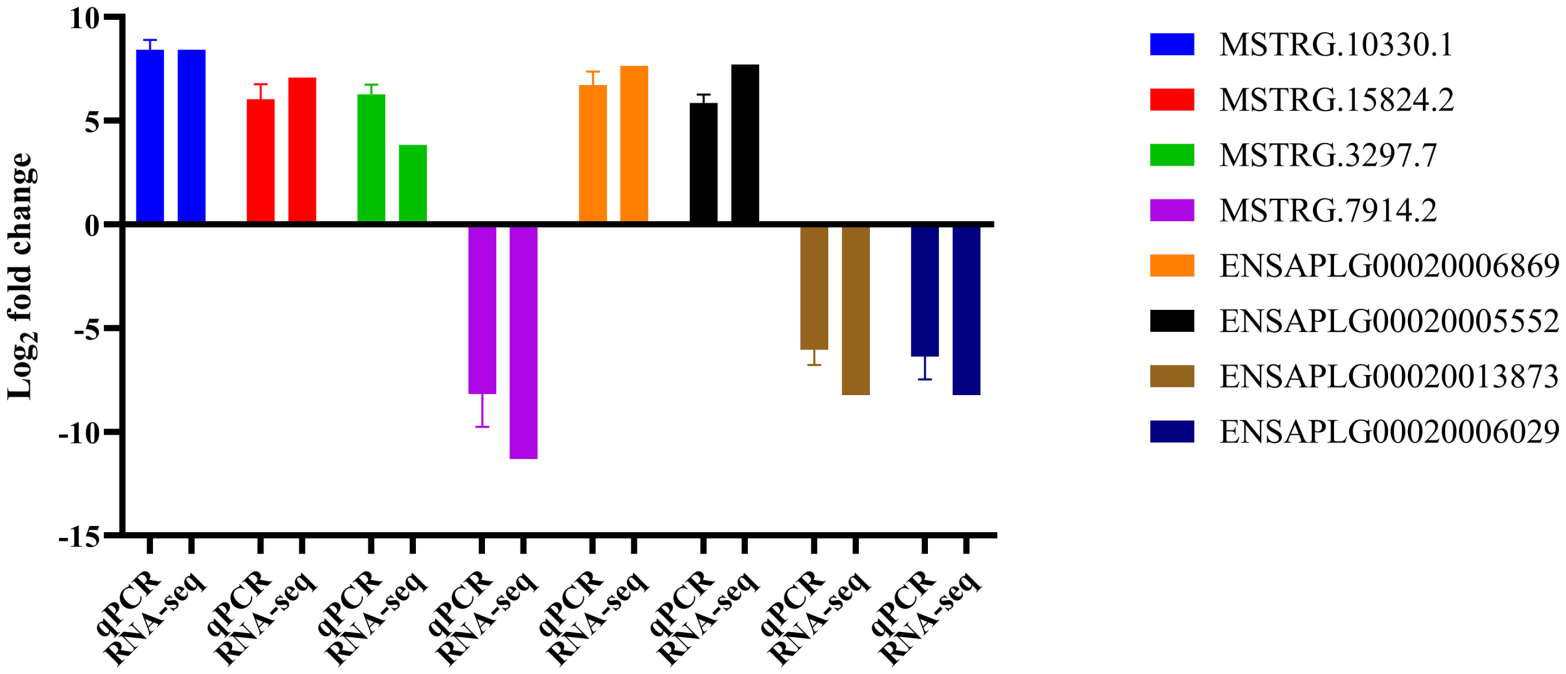
Confirmation of differentially expressed genes by qRT-PCR.

To validate the DEGs identified in our RNA-seq results, we randomly selected 8 DEGs, comprising 4 lncRNAs and 4 mRNAs, for qRT-PCR analysis (Supplementary Table S6). The qRT-PCR analysis revealed that among the 4 lncRNAs, 3 were up-regulated and 1 was down-regulated, with their differential expression patterns aligning with the RNA-seq results. Similarly, the expression trends of the selected mRNAs were also consistent with the RNA-seq analysis (Figure 6).

### Analysis of the basic characteristics of *linc000889*

**Figure 7:**
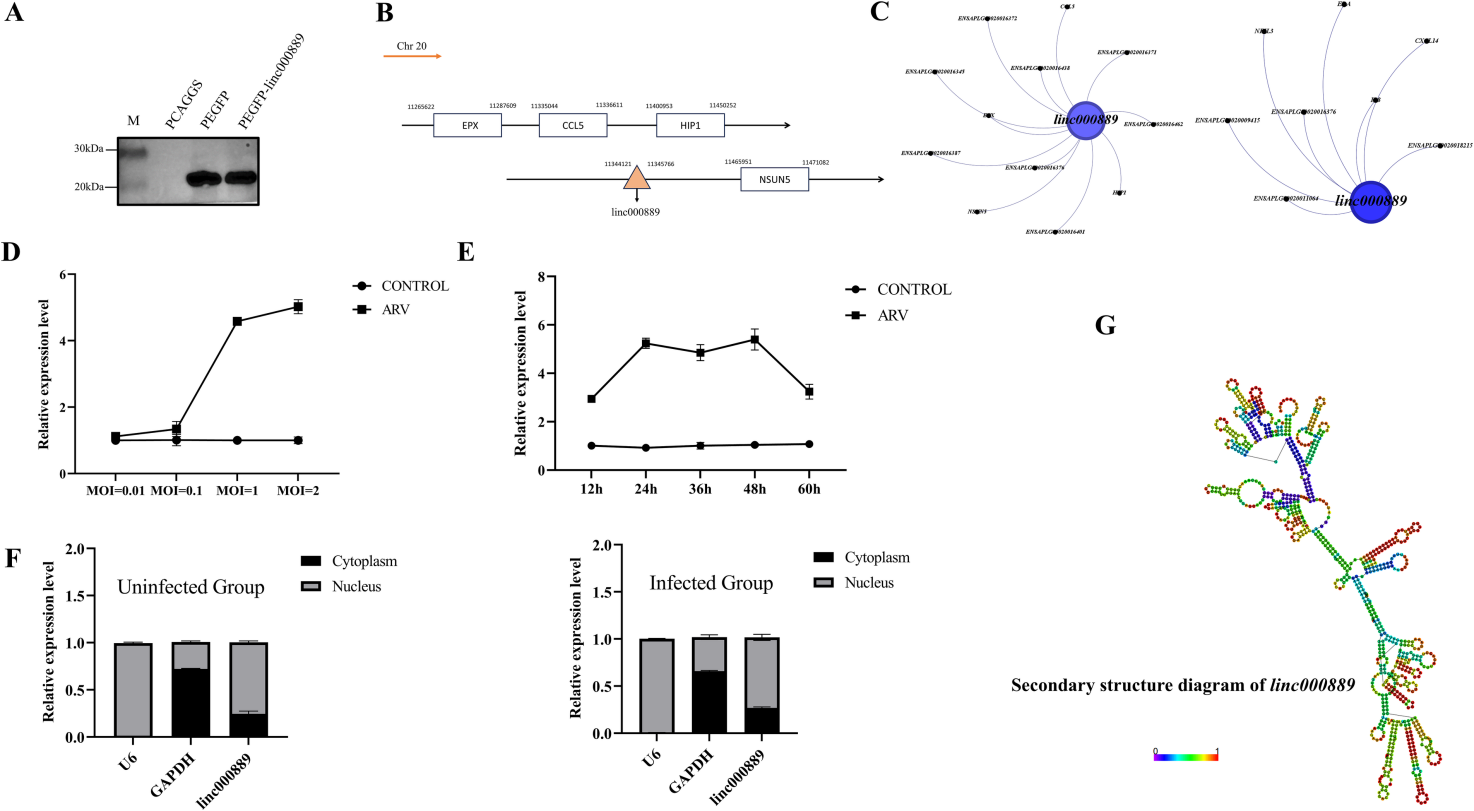
Basic characteristic analysis of *linc000889*. **(A)** Western Blot assay showed that transfection with the PEGFP-linc000889 and PEGFP plasmids resulted in the expression of proteins of the same size. **(B)** The location of *linc000889* in the duck genome. **(C)** Gephi maps the cis-target gene and trans-target gene relationships of *linc000889* related to immune functions. **(D)** qRT-PCR to detect the expression changes of *linc000889* under different MOI conditions at 36 h. **(E)** qRT-PCR to detect the expression changes of *linc000889* under different time conditions at MOI = 1. **(F)** The qRT-PCR verifies the location of *linc000889* in DEF. The left image shows untreated DEF, while the right image shows ARV-infected DEF. **(G)** RNA-fold predicts the secondary structure of *linc000889* (http://rna.tbi.univie.ac.at/cgi-bin/RNAWebSuite/RNAfold.cgi). Each experiment was repeated three times. The data are the means ± SEM (*n* = 3).

From the sequencing data, we identified 43 lncRNAs with log_2_ fold changes greater than 6 and lengths ranging from 200 to 3,000 nucleotides, which were up-regulated (Supplementary Table S7). To further narrow down our focus, we conducted a rigorous screening process to select lncRNAs that were enriched in common immune pathways. Following this, we performed fragment amplification to identify our experimental subject, *linc000889*. Analyses using CNCI, Pfam-Scan, and PIEK indicated that *linc000889* lacked the potential to encode proteins. In addition, western blot results showed that the PEGFP vector group inserted with *linc000889* (PEFGP-linc000889) exhibited the same protein size as the PEGFP group, confirming that *linc000889* does not encode proteins or small peptides (Figure 7A). We further examined the genomic location of *linc000889* (Figure 7B), finding it located on the negative strand of chromosome 20 in ducks. We determined that it is an intergenic lncRNA, transcribed from a gap sequence between genes encoded in the genome, also known as a long intergenic non-coding RNA (LincRNA). Since this lincRNA has not been previously reported in ducks, we named it *linc000889*. Furthermore, we identified the cis-target genes of *linc000889*, including *CCL5*, *HIP1*, and *EPX*, among others, as well as the trans-target genes associated with immune responses, such as *IL8*, *CXCL14*, and *EDA*, among others (Figures 7C). Our study demonstrated that the expression level of *linc000889* exhibited dose-dependent variations in response to different infection doses of ARV at 36 h post DEF infection (Figure 7D). Notably, when the MOI is 1, the expression pattern of *linc000889* over time is not distinctly discernible. The data indicates that the expression of *linc000889* peaks at 48 h and then gradually diminishes (Figure 7E). Employing lncLocator 2.0,^4^ we predicted the cellular localization of *linc000889*, and the results suggested that it is predominantly localized to the nucleus. To substantiate this prediction, we performed the isolation of nuclear and cytoplasmic fractions and extracted RNA for qRT-PCR analysis. Consistent with the bioinformatics prediction, the qRT-PCR results confirmed that *linc000889* is primarily distributed in the nucleus (Figure 7F). This finding establishes a foundation for our forthcoming investigations into the mechanistic role of *linc000889*. We predicted the secondary structure of *linc000889* using RNAfold^5^ (Figure 7G), and the results indicated that it possesses multiple hairpin structures, suggesting that *linc000889* may have significant functional potential.

### *Linc000889* inhibits the replication of ARV

**Figure 8:**
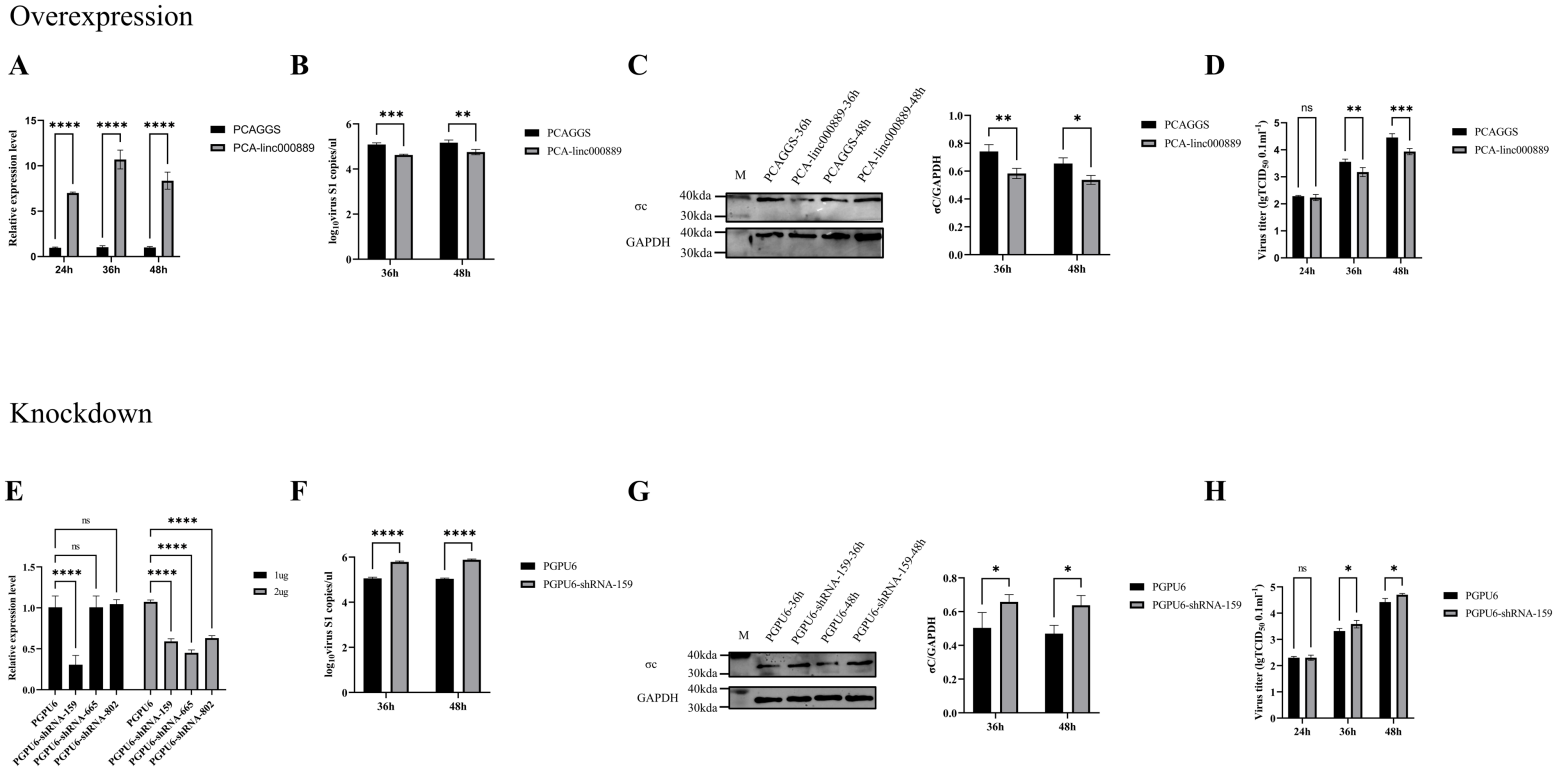
*Linc000889* was shown to inhibit ARV replication through both overexpression and knockdown experiments. **(A)** Verify the expression of PCA-linc000889 in DEF. Transfect 2 μg of control plasmid PCAGGS and experimental plasmid PCA-linc000889 into 12-well plates, collect RNA samples at 24 h, 36 h, and 48 h, and use qRT-PCR to detect the transcriptional changes of *linc000889* after overexpression. **(B–D)** Overexpression experiment: Transfect 2 μg of the experimental plasmid (PCA-linc000889) and control plasmid (PCAGGS) into 12-well cell plates. After 24 h, infect the cells with ARV at an MOI of 0.5. Collect RNA, protein, and viral suspension samples at corresponding time points for qRT-PCR, western Blot, and TCID_50_ detection to analyze changes in viral transcription, translation, and titer. **(E)** Transfect 1 μg or 2 μg of the control plasmid PGPU6 and three experimental plasmids: PGPU6-shRNA-159, PGPU6-shRNA-665, and PGPU6-shRNA-802 into 12-well plates, collect RNA samples at 24 h, 36 h, and 48 h, and use qRT-PCR to detect changes in *linc000889* transcription levels. **(F–H)** Knockdown experiment: Transfect 1 μg of the experimental shRNA PGPU6-shRNA-159 and the control shRNA PGPU6 into 12-well cell plates. After 24 h, infect the cells with ARV at an MOI of 0.5. Collect RNA, protein, and viral suspension samples at corresponding time points for qRT-PCR, western Blot, and TCID_50_ analysis to assess changes in viral transcription, translation, and titer levels. Each experiment was repeated three times, and data are presented as mean ± SEM (*n* = 3). Statistical significance: ns, *p* > 0.05; *p < 0.05; **p < 0.01; ***p < 0.001; ****p < 0.0001.

To elucidate the impact of *linc000889* on viral replication, we engineered an expression vector, PCA-linc000889. The transfection efficiency of PCA-linc000889 was assessed using qRT-PCR (Figure 8A). The results indicated that the PCA-linc000889 transfected group exhibited significantly higher *linc000889* expression levels compared to the PCAGGS transfected group. Following the overexpression of *linc000889* for 24 h, ARV was introduced at an MOI of 0.5, and subsequent collection of RNA and protein samples was performed. qRT-PCR data revealed a significant reduction in ARV-S1 genomic copy numbers at 36 h and 48 h post-infection (Figure 8B), and western blot analyses also indicated a substantial decrease in the protein levels of ARV-σc (Figure 8C). Subsequently, we analyzed the viral titer results and observed a decrease in viral load within the group transfected with PCA-linc000889 (Figure 8D). To ascertain whether *linc000889* influences viral replication by affecting cell viability, we conducted CCK8 assays, which revealed no significant difference in cell viability between the experimental and control groups (Supplementary Figure S2A).

To further assess the role of *linc000889* in viral replication, we transfected 1 μg or 2 μg of shRNA (PGPU6, PGPU6-shRNA-159, PGPU6-shRNA-665 and PGPU6-shRNA-802) in 12-well plates. The results demonstrated that *linc000889* exhibited significant knockdown when transfected with 1 μg of PGPU6-shRNA-159. After transfecting with shRNA159 for 24 h, ARV infection was initiated at an MOI of 0.5, and RNA and protein samples were collected at 36 h and 48 h post-infection. QRT-PCR and western blot analyses demonstrated that the knockdown of *linc000889* significantly increased ARV-S1 copy numbers and ARV-σc protein expression levels (Figures 8F,G). Viral titer measurements also confirmed an increase in viral titer within the shRNA159-transfected group (Figure 8H). CCK8 assay results again showed no significant difference in cell viability between the experimental and control groups (Supplementary Figure S2B). Collectively, these findings lead us to conclude that *linc000889* exerts an inhibitory effect on the replication of ARV.

## Discussion

Transcriptome sequencing has rapidly developed into a critical technique in molecular biology for elucidating gene expression patterns and identifying novel transcripts, making significant contributions to disease diagnosis, treatment development, and public health research (Zhou et al., 2017; Uphoff et al., 2019; Yin et al., 2020). In our study, we utilized transcriptomic sequencing to identify 8,391 DE mRNAs and 817 DE lncRNAs. These findings are expected to offer deeper insights and serve as a valuable reference for the prevention and treatment of ARV at the molecular biological level.

We analyzed the DE mRNAs from the transcriptome data and found that Polymerase 9 (PARP9) was down-regulated in this study. *PARP9*, a member of the *PARP* family, enhances type I interferon (IFN) responses to RNA viruses by binding to viral RNA and activating the PI3K and AKT3 pathways (Xing et al., 2021). *TRIM33* can inhibit viral replication by degrading viral proteases that target viral integrases (Ali et al., 2019). In our study, we discovered that *TRIM33* exhibited an up-regulation trend, which suggested that it might have played a similar role in the ARV infection process in ducks. Toll-like receptor 7 (TLR7) is a crucial member of the Toll-like receptor family and it was up-regulated in this study. Primarily responsible for recognizing and responding to RNA virus infections, *TLR7* plays a key role in regulating both innate and adaptive immune responses. Nanoparticle adjuvants, with *TLR7* as their main component, have been reported to significantly enhance immune responses against influenza and severe acute respiratory syndrome coronavirus type 2 (Yin et al., 2023). Moreover, studies have demonstrated *TLR7*’s significant regulatory role in host infection by rabies virus (Luo et al., 2020), Newcastle disease virus (Zhang et al., 2018), HIV (Meng et al., 2021), and others (Luo et al., 2017; Solmaz et al., 2019). Similarly, the *TRIM* family gene *TRIM29* exhibited differential expression in our experiment and was down-regulated, potentially impacting the host’s innate immunity. *TRIM29* can target *NLRP6* and *NLRP9b* to modulate intestinal inflammation (Wang et al., 2024b), mitigate viral myocarditis by attenuating PERK-driven ER stress (Wang et al., 2024a), and negatively regulate type I IFN responses to RNA viruses (Xing et al., 2018). Furthermore, *TRIM29* can enhance DNA virus infections by inhibiting respiratory tract immunity (Xing et al., 2016; Xing et al., 2017). In cancer biology, *linc00324* suppresses *TRIM29* via *miR-195-5p* upregulation, inhibiting thyroid papillary carcinoma proliferation and invasion (Xu et al., 2020), while *ELFN1-AS1* promotes gastric cancer progression by suppressing *miR-211-3p* and upregulating *TRIM29* (Huang et al., 2023). And we identified a large number of *DUSP* family genes that were up-regulated and down-regulated, including *DUSP19*, *DUSP28*, *DUSP12*, *DUSP7*, *DUSP14*, *DUSP16*, *DUSP5*, *DUSP15*, *DUSP23*, *DUSP4*, and *DUSP26*. The *DUSP* family plays a key role in cell signal transduction by regulating the MAPK and JNK signaling pathways through dephosphorylation (Ha et al., 2019; Sun et al., 2021). MicroRNA *Gga-miR-30c-5p* can inhibit the ARV-induced autophagy process by targeting the *ATG5* gene, thereby effectively inhibiting ARV replication (Zhou L. et al., 2022). Similarly, *ATG5* was up-regulated in this study. In addition, the levels of differentially expressed actin-related factors also changed significantly. Previous studies have shown a close interaction between actin and viral infection (Miazza et al., 2011; Newsome and Marzook, 2015).

Of course, lncRNA also has enormous research value. LncRNA can play an important role in the process of viral infection, promoting or inhibiting viral replication by binding to DNA, RNA, or protein (Xiao et al., 2021; Hu et al., 2023; Zhao et al., 2023). It was reported that lncRNA *IALNCR* expression in host cells reduces MAPK8/JNK1 expression, indirectly activates *caspase-3*, leading to cell-autonomous death and thereby inhibiting BVDV replication (Gao et al., 2022). LncRNA *MAHAT* can bind to DDX6, preventing DDX6 from binding to ZNF34, thereby controlling the expression of ZNF34, enhancing the release of type I interferon, and inhibiting the spread of PRRSV (Liu Y. et al., 2022). LncRNA *SAAL* prevents the replication of influenza virus IAV by increasing the transcriptional levels of Serpina 3i and the mRNA levels of IFN-β and ISGs (Liu Q. et al., 2022). *Lnc-000641* increases PRV replication by inhibiting the JAK/STAT 1 pathway (Fang et al., 2021). In this study, we identified a functional lncRNA, *linc000889*, which exhibits the capacity to suppress ARV replication. We conducted a systematic study to ascertain the impact of *linc000889* on the transcription, translation, and virulence of ARV. Our findings revealed that *linc000889* markedly repressed ARV-*S1* transcription, σc protein expression, and viral titer. Based on the predicted target genes and associated pathways for *linc000889*, we speculate that the inhibitory effect on ARV replication may be mediated through the NOD-like receptor signaling pathway (NLR), the Toll-like receptor (TLR) pathway, or the RIG-I-like receptor (RLR) pathway. We made this hypothesis because the cis-target gene *CCL5* and the trans-target gene *IL-8* of *linc000889* showed significant enrichment in the NLR, RLR, and TLR pathways according to KEGG pathway analysis. The RLR family, comprising retinoic acid-inducible gene I and melanoma differentiation-associated protein 5, serves as a pivotal sensor for detecting pathogen-associated molecular patterns. Upon recognition of viral double-stranded RNA by host, these receptors initiate the synthesis of IFN and other pro-inflammatory cytokines, which in turn triggers a downstream signaling cascade, ultimately activating an antiviral immune response (Rehwinkel and Gack, 2020). For instance, *Lnc-Lsm3b* can competitively bind to exogenous RNA viruses, modulating RIG-I conformation and consequently inhibiting RIG-I-mediated signaling pathways, which reduces IFN-I production and helps maintain immune homeostasis (Jiang et al., 2018). LncRNA *NEAT1* can play an important role in the pathogenesis of acute kidney injury by activating the NLRP3 inflammasome (Xue et al., 2024). NLRs are not only involved in inflammation-related pathways, but recent studies have also uncovered their novel roles in antiviral innate immune signaling (Zheng, 2021). LncRNAs can also act through the TLR pathway, for example, *lncRNA-CR33942* activates the TLR pathway by interacting with the Dorsal-related immunity factor, inducing the expression of antimicrobial peptides, and thereby enhancing immune defense against pathogens (Zhou H. et al., 2022).

Both mRNA vaccines and lncRNA-based drug development are currently topics of significant interest. Beyond the widely utilized COVID-19 mRNA vaccines (Kohli et al., 2024), mRNA vaccines targeting a range of infectious diseases, including influenza, HIV, and RSV, are actively in development (Zhang et al., 2023; Allen and Ross, 2024; Kutikuppala et al., 2024; Liu et al., 2024). Concurrently, lncRNA-derived peptides have demonstrated not only immunogenicity but also the capacity to elicit a potent anti-tumor response, underscoring the substantial therapeutic potential of lncRNA in oncology (Barczak et al., 2023).

In summary, our study has successfully screened numerous DE mRNAs and lncRNAs induced by ARV. Notably, these include genes such as *PARP9*, *TLR7*, *TRIM33*, and *ATG5*, which have been previously reported to play important roles in immune regulation. Given the crucial functions of these genes, we hypothesize that they also exert indispensable effects during the replication of ARV. More significantly, we have successfully screened a novel lncRNA, *linc000889*, and we have demonstrated that it can inhibit ARV replication. This interesting discovery has laid a solid groundwork for future investigations into the molecular mechanisms underlying *linc000889*’s antiviral activity against ARV.

## Data Availability

The datasets presented in this study can be found in online repositories. The names of the repository/repositories and accession number(s) can be found in the article/Supplementary material.

## Footnotes

^1^ https://daehwankimlab.github.io/hisat2/

^2^ https://geneontology.org/

^3^ https://www.genome.jp/kegg/

^4^ http://www.csbio.sjtu.edu.cn/bioinf/lncLocator2/

^5^ http://rna.tbi.univie.ac.at/cgi-bin/RNAWebSuite/RNAfold.cgi
