# Supplementary material for: Comprehensive analysis of lncRNAs and mRNAs revealed potential participants in the process of avian reovirus infection
Source: Front Microbiol. 2025 Feb 5;16:1539903. doi: 10.3389/fmicb.2025.1539903 (PMC11835999; doi:10.3389/fmicb.2025.1539903)
Supplement: Supplementary file 1 [file Table_1.docx]

**Supplementary Table1 Standard curve of S1 gene of ARV**

Standard curve: S1 gene copy number = (40.725 - Cq value) / 3.525

qPCR reaction procedure

| Step | Duration | Temperature | Cycle |
| --- | --- | --- | --- |
| predenaturation | 30s | 95℃ | 1 |
| denaturation | 10s | 95℃ | 40 |
| annealing | 10s | 55℃ |  |
| extension (Fluorescence acquisition) | 30s | 72℃ |  |
| dissolution | Instrument default | Instrument default | 1 |

qPCR reaction system

| Substance | Volume |
| --- | --- |
| SYBR mix | 10.0ul |
| Forward primer | 1.0µl |
| Reverse primer | 1.0µl |
| cDNA | 2.0µl |
| ddH_2_O | 6.0µl |
