# Supplementary material for: Comprehensive analysis of lncRNAs and mRNAs revealed potential participants in the process of avian reovirus infection
Source: Front Microbiol. 2025 Feb 5;16:1539903. doi: 10.3389/fmicb.2025.1539903 (PMC11835999; doi:10.3389/fmicb.2025.1539903)
Supplement: Supplementary file 2 [file Table_2.docx]

**Supplementary Table2 RNA-Seq Map Statistics**

| Sample | Clean Reads | Total Mapped | Multiple Mapped | Uniquely Mapped |
| --- | --- | --- | --- | --- |
| DEF_c1 | 79447618 | 72775240 (91.60%) | 8011574 (11.01%) | 64763666 (88.99%) |
| DEF_c2 | 85812788 | 78077252 (90.99%) | 8630660 (11.05%) | 69446592 (88.95%) |
| DEF_c3 | 72506824 | 66311178 (91.46%) | 5141504 (7.75%) | 61169674 (92.25%) |
| DEF_v1 | 74608406 | 49729113 (66.65%) | 1183466 (2.38%) | 48545647 (97.62%) |
| DEF_v2 | 77392028 | 52519464 (67.86%) | 1694643 (3.23%) | 50824821 (96.77%) |
| DEF_v3 | 64902482 | 43507884 (67.04%) | 1073848 (2.47%) | 42434036 (97.53%) |
