# Supplementary figures and images for: Comprehensive analysis of lncRNAs and mRNAs revealed potential participants in the process of avian reovirus infection
Source: Front Microbiol. 2025 Feb 5;16:1539903. doi: 10.3389/fmicb.2025.1539903 (PMC11835999; doi:10.3389/fmicb.2025.1539903)

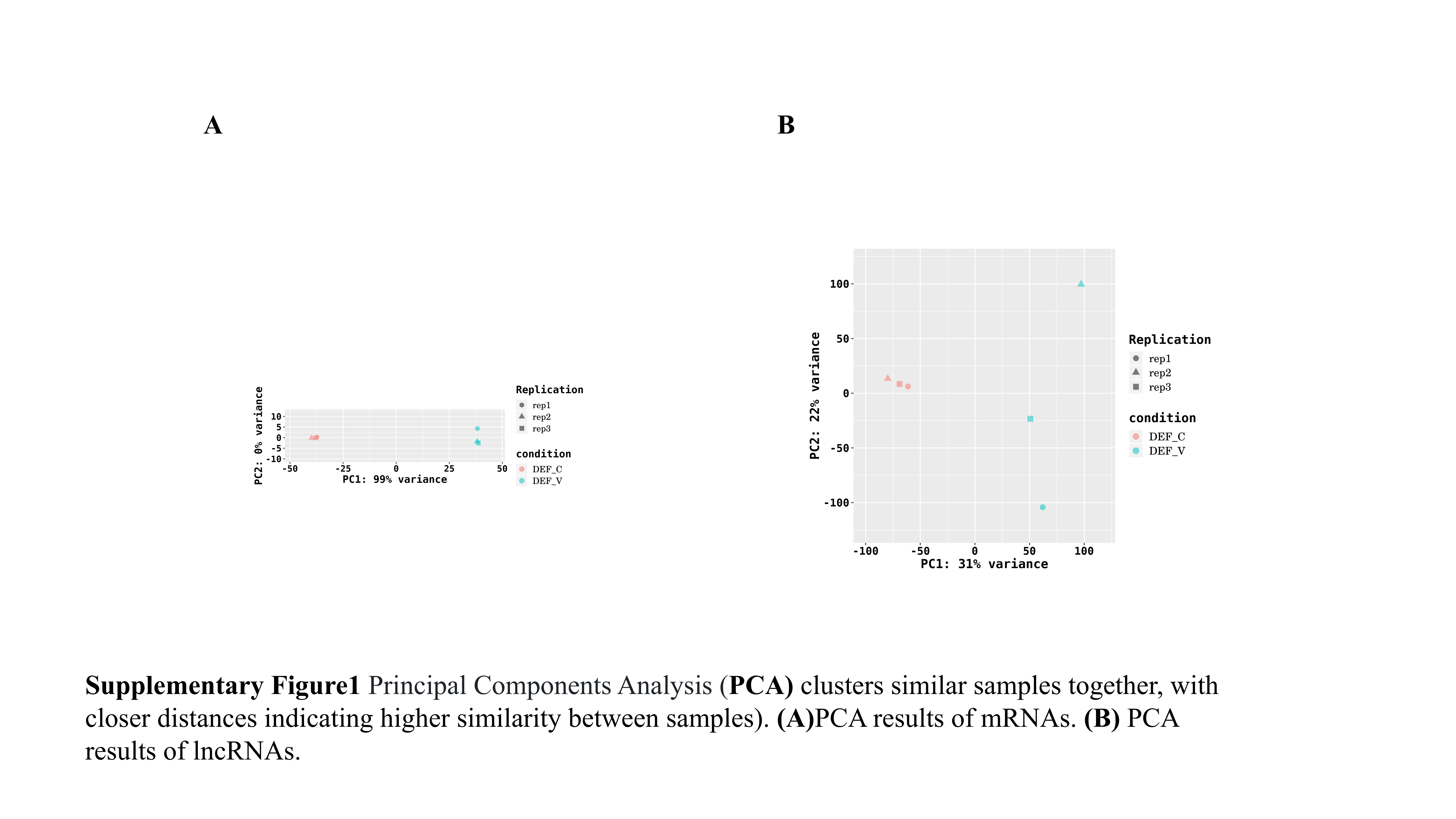

Supplement: Supplementary file 8 [file Image_1.tif]

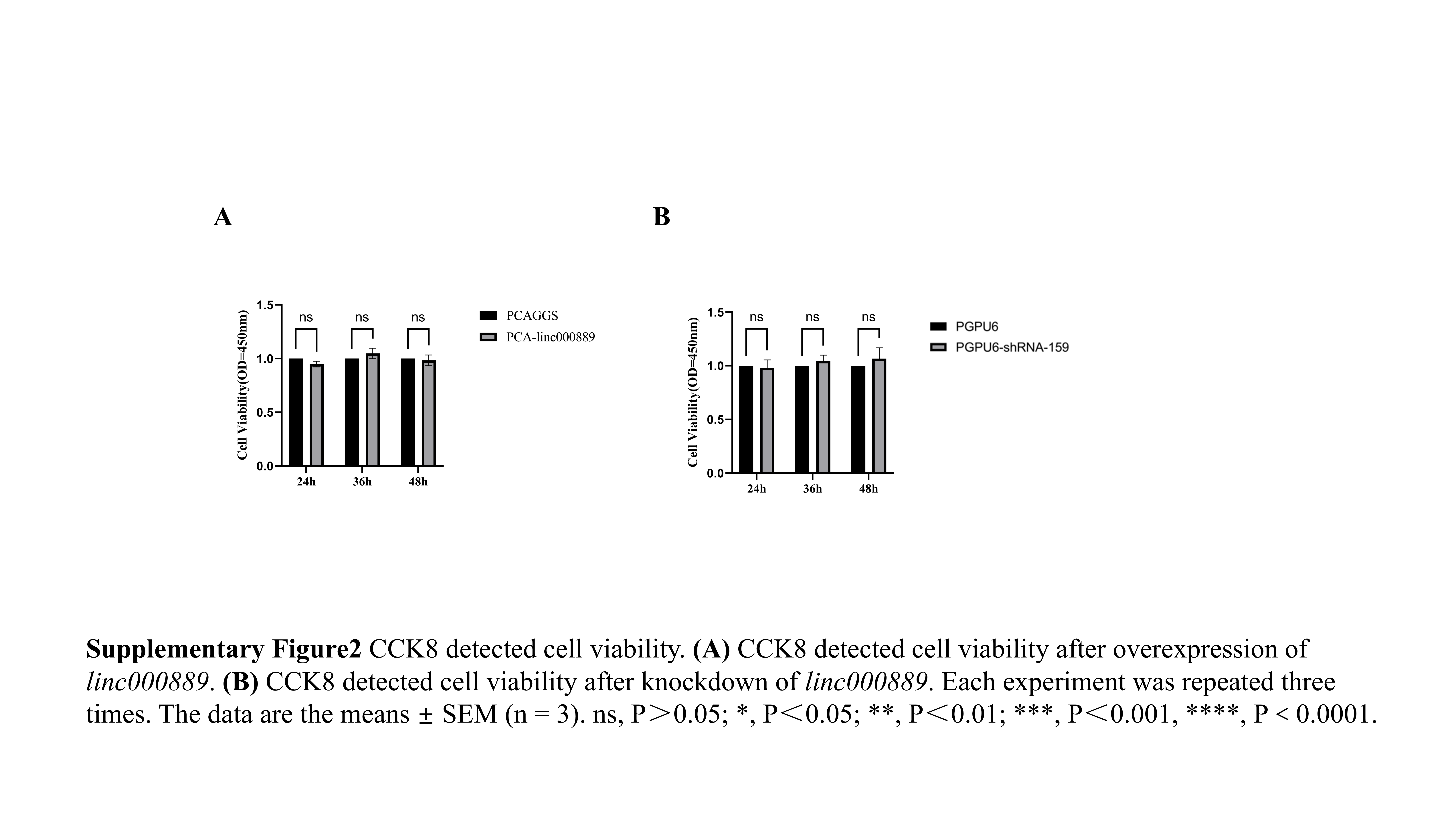

Supplement: Supplementary file 9 [file Image_2.tif]
